# Robust PCA with *L*_*w*,∗_ and *L*_2,1_ Norms: A Novel Method for Low-Quality Retinal Image Enhancement

**DOI:** 10.3390/jimaging10070151

**Published:** 2024-06-21

**Authors:** Habte Tadesse Likassa, Ding-Geng Chen, Kewei Chen, Yalin Wang, Wenhui Zhu

**Affiliations:** 1 Department of Biostatistics, College of Health Solutions, Arizona State University, Phoenix, AZ 85004, USA; 2 Department of Statistics, University of Pretoria, Pretoria 0028, South Africa; 3 Computer Science and Engineering, School of Computing and Augmented Intelligence, Arizona State University, Phoenix, AZ 85287-8809, USA

**Keywords:** RPCA, *τ_i_*, *L*_*w*,∗_ norm, *L*_2,1_ norm, image enhancement

## Abstract

Nonmydriatic retinal fundus images often suffer from quality issues and artifacts due to ocular or systemic comorbidities, leading to potential inaccuracies in clinical diagnoses. In recent times, deep learning methods have been widely employed to improve retinal image quality. However, these methods often require large datasets and lack robustness in clinical settings. Conversely, the inherent stability and adaptability of traditional unsupervised learning methods, coupled with their reduced reliance on extensive data, render them more suitable for real-world clinical applications, particularly in the limited data context of high noise levels or a significant presence of artifacts. However, existing unsupervised learning methods encounter challenges such as sensitivity to noise and outliers, reliance on assumptions like cluster shapes, and difficulties with scalability and interpretability, particularly when utilized for retinal image enhancement. To tackle these challenges, we propose a novel robust PCA (RPCA) method with low-rank sparse decomposition that also integrates affine transformations $\tau_{i}$, weighted nuclear norm, and the $L_{2,1}$ norms, aiming to overcome existing method limitations and to achieve image quality improvement unseen by these methods. We employ the weighted nuclear norm $\left(L_{w,*}\right)$ to assign weights to singular values to each retinal images and utilize the $L_{2,1}$ norm to eliminate correlated samples and outliers in the retinal images. Moreover, $\tau_{i}$ is employed to enhance retinal image alignment, making the new method more robust to variations, outliers, noise, and image blurring. The Alternating Direction Method of Multipliers (ADMM) method is used to optimally determine parameters, including $\tau_{i}$, by solving an optimization problem. Each parameter is addressed separately, harnessing the benefits of ADMM. Our method introduces a novel parameter update approach and significantly improves retinal image quality, detecting cataracts, and diabetic retinopathy. Simulation results confirm our method’s superiority over existing state-of-the-art methods across various datasets.

## 1. Introduction

Estimating genuine low-rank components from corrupted high dimensional images is a significant advancement in health applications, particularly in biomedical image processing. Leveraging these inherent low-rank characteristics in biomedical image processing is crucial for transforming a wide array of real-world health-related applications, fostering substantial progress and innovation in the field. Among these, image enhancement has garnered significant attention from researchers, particularly in fields such as cataract diagnosis [1,2,3,4], cancer detection [5], retinal enhancement [6,7,8,9], medical image segmentation [10,11,12], scene categorization [13], crime detection [14], sparse coding [15], image denoising [16], communications and computational imaging [17], computer vision [18], and retinal disease detection [19,20] such as glaucoma [21,22]. However, the existing methods encounter significant challenges such as image blurring, noise, and corruptions in biomedical imaging.

Nowadays, deep learning methods have been widely applied to biomedical image processing for medical diagnosis [23,24]. An example is the deep hybrid network low-light image enhancement approach via a unified network with two different streams to capture the global content and the salient structures of the clear image [25]. Yet, this method requires a large volume of training data and storage to process millions of parameters. A hybrid retinal image enhancement algorithm was proposed by [26] for detecting diabetic retinopathy and improving the low quality, using the deep learning model. However, this method is computationally expensive and lacks robustness when the percentage of noise level in images is high. To improve poor-quality retinal fundus images, a simple but effective end-to-end unsupervised learning framework was proposed by [7]. Moreover, Zhu et al. (2023) [27] introduced an unpaired image-to-image translation method for converting low-quality images into their high-quality counterparts. Similarly, Liu et al. (2022) [28] proposed the pyramid constraint to create a degradation-invariant supervised learning enhancement network (PCE-Net). This approach reduces the need for clinical data and effectively enhances the hidden intrinsic dataset. However, challenges still persist in clinical scenarios. To address the challenges of uneven illumination, blurring, and various anomalies, in enhancing retinal images, Liu and Huang [29] introduced a combined approach for improving low-quality retinal images and segmenting blood vessels, utilizing a diffusion model for both tasks [30]. Furthermore, Oh et al. (2023) [31] introduced a novel retina image enhancement framework using scattering transform. This framework entails training an enhancement model that relies on paired images to convert low-quality images into their high-quality counterparts. However, these methods lack generalization on data outside of the training set and encounter problems with mode collapse with the GAN-based unsupervised method, including difficulties in optimizing the parameters. Additionally, deep learning methods via a new frontier of machine learning require more training [32], which takes more computational time [33,34], and have poor real-world clinical generalizability, limiting their practicality in medical imaging [35,36]. Moreover, deep learning models are often considered black boxes, lacking the interpretability crucial for clinical acceptance [37,38]. Hence, it is essential to consider traditional unsupervised machine learning methods to enhance the quality of retinal images.

To overcome the drawbacks of the deep learning methods, unsupervised learning methods have been proposed for retinal image processing [39]. For instance, a contrast-limited adaptive histogram equalization (CLAHE) method was proposed by [40], and a histogram equalization method (HEM) incorporating a tunable parameter was proposed by [41]. However, these methods fail to maintain image quality, often resulting in excessive blurring of the edges. In an effort to enhance retinal image quality, researchers have proposed the low-light image enhancement method (LLIEM). This innovative approach incorporates multi-resolution branches to gain a deeper understanding of diverse levels of local and global context through distinct streams as outlined in reference [42]. However, this method suffers loss of information and semantic content. The machine learning technique was proposed by [43] for retinal image enhancement and glaucoma detection—review and perspective—and a hybrid image enhancement algorithm (HIEA) was developed by [44], which incorporates a median filter for image denoising and is also time consuming. Although these methods do not require training datasets, they lack robustness in high-dimensional medical images with noisy data. To enhance the low-quality of images, a spatial domain filtering method incorporated with the $L_{w,*}$ norm by [45] and a new approach proposed by [46] suggest detecting microaneurysms by considering grey-scale transformations that reduce spatial dependence between images as in [47]. Gao et al. (2019) [48] applied adaptive retinal mechanisms to enhance fundus images as demonstrated by [49,50]. To enhance the quality of retinal images, several methods [51] have been proposed. However, these approaches require explicit training data and exhibit more computational complexity.

Recently, Jiang et al. (2023) [52] proposed an event-based low-illumination image enhancement technique. However, these methods lack the ability to accurately estimate the true underlying objects and reduce nonexistent blurring when enhancing the low-quality color fundus images. This affects diagnostic accuracy and hinders their direct application to fundus images. Given the constraints of conventional unsupervised learning techniques, there is a compelling need to introduce a novel method capable of robust generalization and effective noise handling in high-dimensional and complex image datasets.

To address the limitations of unsupervised learning methods in terms of robustness, interpretability, and computational efficiency for various imaging tasks, numerous methods have been developed ever since the epoch-making emergence of Robust Principal Component Analysis (RPCA) by [53,54]. These methods, proposed by [53], and a myriad of other methods [55,56], proposed to enhance the quality of images through robust low-rank-sparse image representation. For instance, Wright et al. (2009) and Kopriva et al. (2016) [57,58] demonstrated the potential of low-rank matrix approximation to enhance the quality of images. However, the full benefits of these techniques have yet to be extensively explored in the realm of biomedical imaging. In this regard, the low-rank approximation model [59,60,61] has been explored with great success in natural image recovery. The ADMM approach is employed to iteratively update optimization variables, similar to the work of [61,62,63]. However, while the performance of these methods appears promising, the methods assign the same singular values to different images, which affects the performance in complex and highly correlated images. Similarly, tensor low-rank representation (TLLR) for image denoising was proposed by [64], while [8,65] proposed sparse rank-constrained learning and its application for medical image grading. However, they fail to denoise highly correlated images, as they do not consider the $L_{2,1}$ and $L_{w,*}$ norms, which undermines the method’s performance.

Despite its theoretical foundation and practical efficacy, the RPCA methodology put forth by [9,53,61,62,64,66] fails to differentiate between singular values in image data, employing a uniform approach to regularization across all singular values. This approach leads to an inaccurate estimation of the low-rank component of image data [53,67,68]. To overcome this issue, this paper proposes a novel RPCA method by adding $\tau_{i}$, $L_{w,*}$ and $L_{2,1}$ norms. To be robust against the adverse effects, the new method combines $\tau_{i}$, $L_{w,*}$ and $L_{2,1}$ norms for a better biomedical image processing. To reduce the misalignment problem in retinal image recovery, affine transformations are incorporated to render more accurate robust image enhancement. Our method benefits from the weighted nuclear norm, a norm which assigns varying weights to different retinal images through singular value decomposition, enhancing its adaptability and effectiveness. In this paper, an alternative novel method is proposed which is robust to the selection of different EyeQ and cataract images taken from the Kaggale datasets. The ADMM technique is considered, and a new set of equations is formed to estimate the optimization parameters and affine transformations in an iterative process. The simulation results demonstrate that the proposed method outperforms state-of-the-art techniques for enhancing retinal images on certain popular available datasets.

The key contributions of this paper can be summarized as follows.

(1) In this paper, we proposed a novel RPCA that integrates affine transformations to iteratively and accurately estimate the low-rank component from highly complicated retinal images. This work incorporates affine transformations to rectify distorted or misaligned retinal images, aiming to achieve improved quality. As a result of incorporating affine transformation, a new updated parameter is achieved. To tackle the computational load, all parameters are individually solved using ADMM and then updated iteratively in a round-robin manner.

(2) The novel approach aims to enhance robustness against diverse adverse effects, such as measurement noise, image blurring, and artifacts by integrating the previously unexplored $L_{w,*}$ and $L_{2,1}$ norms in retinal imaging enhancement techniques. In this work, the $L_{w,*}$ norm is employed to assign weights to singular values for each retinal image, providing essential adaptability for scenarios where specific features or dimensions need emphasis during decomposition. Additionally, the $L_{2,1}$ norm is utilized to effectively eliminate correlated samples and outliers within complex retinal images, and it enables denoising, feature highlighting, and artifact removal from retinal images, resulting in clearer and more informative images, beneficial for medical diagnosis and analysis.

(3) The developed method is efficiently solved using the ADMM approach iteratively, ensuring robustness and effectiveness in addressing the complexities of retinal image enhancement.

(4) The method’s effectiveness is demonstrated through extensive simulations with multiple retinal images, showing improved image quality by addressing degradation factors such as cataract, glaucoma and diabetic retinopathy in human eyes.

(5) This work not only proposes a novel RPCA method but also aims to draw scholars’ attention to the development of low-rank image representation techniques in retinal, cataract, and cancer imaging, with the goal of reducing anomalies for improved clinical diagnosis in biomedical image processing, an aspect that has not been extensively explored in previous methods, highlighting the potential for advancements in this field.

This paper is structured as follows. Section 2 discusses the novel method of RPCA with $L_{w,*}$ and $L_{2,1}$ norms. We further explain the parameter estimation in the optimization techniques. Section 3 describes the nature of the dataset considered for medical image analysis. Section 4 presents the results of medical image analysis using visualization and numerical analysis. Section 5 provides some discussions and concluding remarks to summarize the paper.

## 2. Methods

Within this section, we describe the development of the new method for retinal image enhancement.

### 2.1. RPCA with $L_{w,*}$ and $L_{2,1}$ Norms

One of the major drawbacks of the existing methods, such as [53,69], is their inefficiency in adequately eliminating outliers and noise, and detecting cataracts, glaucoma, and diseases during biomedical image enhancement in human eyes. To overcome this limitation, the subsequent section introduces a pioneering approach for enhancing retinal images and also detecting cataracts and glaucoma.

### 2.2. The $L_{w,*}$ and $L_{2,1}$ Norms Method

Consider *n* low-quality retinal images, $\left\{R_{i}^{0}\right\}\in {ℜ}^{w\times h\times c},\;i=1,\cdots ,n$, denoting the width and height of the images as *w* and *h*, respectively, with *c* representing the number of channels (e.g., “c = 3” for an RGB image).

Each of these retinal images depicts identical objects and exhibits high correlation with one another. Often, these images are marred by issues such as image blurring due to various adverse annoying effects. Then, it is possible to stack these images into a matrix: $N=\left[vec\left(R_{1}^{0}\right)|vec\left(R_{2}^{0}\right)|\ldots |vec\left(R_{n}^{0}\right)\right]\in {ℜ}^{m\times n}$, as such vec(·) used to denote the vectorization operators for the purpose of stacking images. As such, the original images N can be further decomposed N=L+E [70,71], where $L\in {ℜ}^{m\times n}$ is a clean low-rank or enhanced image, and $E\in {ℜ}^{m\times n}$ denotes a sparse error matrix incurred by outliers or corruptions. The RPCA by [53], which decomposes highly corrupted retinal images as low-rank, can name enhanced image and anomalies as sparse in the form of optimization techniques given by

(1) $$\begin{array}{c} \min_{L,E,\Gamma }{∥L∥}_{*}+\alpha {∥E∥}_{1}\\ s.t\;N=L+E \end{array}$$

where ${∥L∥}_{*}=\sum_{i=1}^{min(m,n)}\sigma_{i}\left(L\right)$, denoting the nuclear norm of the low-rank component matrix L, $\sigma_{i}\left(L\right)$ indicates the singular values of L, ${∥E∥}_{1}$ is the $L_{1}$ norm given by $\sum_{i=1}^{n}\left|E_{i}\right|$, and Γ is the Lagrangian multiplier.

Typically, $R_{i}^{0}$ are usually not well matched, leading to imprecision in the low-rank-sparse decomposition of retinal images after mitigating adverse effects.

To address this, drawing inspiration from [72], we consider $\tau_{i}$ to substantially misaligned retinal images $R_{i}^{0}$ to achieve well-transformed images $R_{i}=R_{i}^{0}o\tau_{i}$, where *o* indicates the transforming operator. Then, by stacking these transformed retinal images into a matrix, we achieve $N_{o\tau }=\left[vec\left(R_{1}\right)|vec\left(R_{2}\right)|\ldots |vec\left(R_{n}\right)\right]\in {ℜ}^{m\times n}=L+E$. Since the solution of $N_{o\tau }$ is intractable due to the nature of the nonlinearity issue, we have to further linearize $N_{o\tau }$. Solving for the parameters related to the constraints $N_{o\tau }=L+E$ is intractable as a result of the nonlinearity issue. To overcome this obstacle, we proceed under the assumption that the alterations induced by these affine transformations $\tau_{i}$ are minor, and an initial $\tau_{i}$ is already available.

Then, we make a linearization to $N_{o\tau }$ by taking the first-order-Taylor-approximation as $N_{o(\tau +\Delta \tau )}\approx N_{o\tau }+\sum_{i=1}^{n}J_{i}\Delta \tau \omega_{i}\omega_{i}^{T}$; as such, $N_{o\tau }\in {ℜ}^{m\times n}$ is denoting a transformed image, $\Delta \tau \in {ℜ}^{p\times n}$ with *p* being the number of variables, $J_{i}=\frac{\partial vec(R_{i}{o\tau }_{i})}{\partial \tau_{i}}\in {ℜ}^{m\times p}$ represents the Jacobian of the *i*-th retinal images with respect to $\tau_{i}$, and $\omega_{i}$ is the standard basis for ${ℜ}^{n}$. Thus, by adding $\tau_{i}$ to N, N is changed to $N_{o\tau }+\sum_{i=1}^{n}J_{i}\Delta \tau \omega_{i}\omega_{i}^{T}$ as in [62,72,73].

To make the proposed method more resilient and robust to noise and outliers, occlusions, blurring and artifacts, the $L_{2,1}$ norms are incorporated by combining the $L_{1}$ into the $L_{2}$ norm, which is employed to manifest the sparsity and the low-rank properties that are regarded as the enhanced retinal images. Also, we transform images and consider the $L_{2,1}$ suggested by [74] to tackle the misalignment problem and highly correlated samples between images. Moreover, the $L_{2,1}$ regularizer is taken into account as the rotational invariant of the $L_{1}$ norm and it captures the collinearity between retinal images which is preferred to overcome and address the lack of robustness due to outliers and anomalies [75].

To boost the performance of the proposed method, and tackle the drawbacks of the nuclear norm [53,59,60,62], the $L_{w,*}$ norm is incorporated to assign weights to singular values in retinal images as demonstrated in [76,77,78]. Subsequently, the overall problem can be formulated as an optimization problem as follows

(2) $$\begin{array}{c} \min_{L,E,\Delta \tau ,\Gamma }{∥L∥}_{w,*}+\alpha {∥E∥}_{2,1}\\ s.t\;N_{o\tau }+\sum_{i=1}^{n}J_{i}\Delta \tau \omega_{i}\omega_{i}^{T}=L+E \end{array}$$

where ${∥L∥}_{w,*}=\sum_{i=1}^{n}\left|w_{i}\sigma_{i}\left(L\right)\right|$, $w_{i}$ is the weight given by $\frac{b/\sqrt{n}}{\sigma i(L)+\varepsilon }$, where b>0 is representing a constant, *n* is the number of similar retinal images in $L_{j}$, $\varepsilon =10^{-16}$ is used to reduce the complexity of dividing by zero, and $\sigma_{i}\left(L\right)$ denotes the singular value of a matrix L [76], then the $L_{2,1}$ norm can be given by ${∥E∥}_{2,1}=\sum_{i=1}^{n}{\left(\sum_{j=1}^{m}E_{ji}^{2}\right)}^{1/2}$ which denotes the $L_{2,1}$ norm of E which denotes the $L_{2,1}$ norm of E [60,62], and Γ and α denote the Lagrangian multiplier and regularization parameter, respectively.

### 2.3. Parameter Estimation

To solve (2), we delve into the augmented Lagrangian function, characterized by:

(3) $$\begin{array}{cc} Ł(L,E,\Delta \tau ,\Gamma ) & ={∥L∥}_{w,*}+\alpha {∥E∥}_{2,1}+\langle \Gamma ,P-L-E\rangle \\ & +\frac{\mu }{2}{∥P-L-E∥}_{F}^{2} \end{array}$$

where $\Gamma \in {ℜ}^{m\times n}$ denotes the Lagrangian multiplier, μ denotes the penalty parameter, and $P=N_{o\tau }+\sum_{i=1}^{n}J_{i}\Delta \tau \omega_{i}\omega_{i}^{T}$. By utilizing an augmented Lagrange multiplier alongside an adaptive penalty as proposed in [79,80], (3) can be reformulated as:

(4) $$\begin{array}{cc} Ł(L,E,\Delta \tau ,\Gamma ) & ={∥L∥}_{w,*}+\alpha {∥E∥}_{2,1}\\ & +\frac{\mu }{2}{∥P-L-E+\frac{\Gamma }{\mu }∥}_{F}^{2} \end{array}$$

Directly solving (4) poses significant computational challenges; thus, we opt for iteratively updating the parameters alternately using the ADMM method [62,81].

Firstly, to update L, we fix E and Δτ as constant, so $L^{\left(k+1\right)}$, updated by

(5) $$L^{\left(k+1\right)}=\underset{L}{\mathrm{argmin}}Ł\left\{\begin{array}{c} L,E^{\left(k\right)},\Delta \tau^{\left(k\right)} \end{array}\right\}$$

*k* is an index representing an iteration. By ignoring all parameters as a constant L, Equation (5) can be rewritten as

(6) $$\begin{array}{cc} L^{\left(k+1\right)} & =\underset{L}{\mathrm{argmin}}Ł\{\alpha {∥L∥}_{w,*}+\frac{\mu^{\left(k\right)}}{2}||P^{\left(k\right)}-L\\ & -E^{\left(k\right)}+\frac{\Gamma^{\left(k\right)}}{\mu^{\left(k\right)}}|{|}_{F}^{2}\} \end{array}$$

Problem (6) is equivalent to the weighted nuclear norm minimization (WNNM) problem [76,81,82], and the closed-form solution of the WNNM operator is given by

(7) $$\begin{array}{c} L=U\hat{\Sigma }V^{T} \end{array}$$

where $L=U\hat{\Sigma }V^{T},\hat{\Sigma }$ is given by $(\mathrm{diag}\left(\sigma_{1}\left(L\right),\ldots ,\mathrm{diag}\left(\sigma_{n}\left(L\right)\right)\right)$ and $\sigma_{i}\left(L\right)=\left\{\begin{array}{c} 0,c_{2}<0\\ \frac{c_{1}+\sqrt{c_{2}}}{2},c_{2}\geq 0 \end{array}\right.$ where $c_{1}=\sigma_{i}\left(K\right)-\varepsilon ,c_{2}={\left(\sigma_{i}\left(K\right)+\varepsilon \right)}^{2}-4C$, ε and *C* are small constants and $K=-\frac{1}{2}\left(E^{k}-P^{k}-\frac{\Gamma^{k}}{\mu^{k}}\right)$.

Secondly, to update E, we keep L and Δτ as constants, then $E^{\left(k+1\right)}$ is updated by

(8) $$\begin{array}{c} E^{\left(k+1\right)}=\underset{E}{\mathrm{argmin}}Ł\left\{\begin{array}{c} L^{\left(k+1\right)},E,\Delta \tau^{\left(k\right)} \end{array}\right\} \end{array}$$

from which E, Equation (8), is reduced as

(9) $$\begin{array}{cc} E^{\left(k+1\right)} & =\underset{E}{\mathrm{argmin}}\{\alpha {∥E∥}_{2,1}+\frac{\mu^{\left(k\right)}}{2}||P^{\left(k\right)}\\ & -L^{\left(k\right)}-E+\frac{\Gamma^{\left(k\right)}}{\mu^{\left(k\right)}}|{|}_{F}^{2}\} \end{array}$$

By considering the lemma as in [83] as constants, the optimal parameter of the *i*-th column of $E^{\left(k+1\right)}$, $E_{j}^{\left(k+1\right)}$ is given by

(10) $$E_{j}^{\left(k+1\right)}=\left\{\begin{array}{cc} \frac{{∥U_{j}^{\left(k\right)}∥}_{2}-\frac{\alpha }{\mu^{\left(k\right)}}}{{∥U_{j}^{\left(k\right)}∥}_{2}}U_{j}^{\left(k\right)}, & \mathrm{if}\;{∥U_{j}^{\left(k\right)}∥}_{2}\geq \frac{\alpha }{\mu^{\left(k\right)}}\\ 0, & \mathrm{otherwise} \end{array}\right.$$

where $U^{\left(k\right)}=\left(P^{\left(k\right)}-L^{\left(k\right)}+\frac{\Gamma^{\left(k\right)}}{\mu^{\left(k\right)}}\right)$, and ${∥.∥}_{2}$ is denoting the Euclidean norm. Next, to optimize Δτ, L and E are considered fixed, and then $\Delta \tau^{\left(k+1\right)}$ is given by

(11) $$\Delta \tau^{\left(k+1\right)}=\underset{\Delta \tau }{\mathrm{argmin}}Ł\left\{\begin{array}{c} L^{\left(k+1\right)},E^{\left(k+1\right)},\Delta \tau \end{array}\right\}$$

Consider all other parameters independent of Δτ as constant, from which we can obtain

(12) $$\begin{array}{cc} \Delta \tau^{\left(k+1\right)}=\underset{\Delta \tau }{\mathrm{argmin}} & \{\frac{\mu^{\left(k\right)}}{2}||P^{\left(k\right)}-L^{\left(k+1\right)}\\ & -E^{\left(k+1\right)}+\frac{\Gamma^{\left(k\right)}}{\mu^{\left(k\right)}}|{|}_{F}^{2}\} \end{array}$$

Solving (12) by considering the thresholds operator as in [72], we can achieve an optimal parameter given by

(13) $$\begin{array}{cc} {\Delta \tau }^{\left(k+1\right)}=\sum_{i=1}^{n} & J_{i}^{+}(L^{\left(k+1\right)}+E^{\left(k+1\right)}-\\ & -N_{o\tau }-\frac{\Gamma^{\left(k\right)}}{\mu^{\left(k\right)}})\omega_{i}\omega_{i}^{T} \end{array}$$

where $J_{i}^{+}$ denotes the Moore–Penrose pseudoinverse of $J_{i}$ [84]. Following the same procedure as above, the Lagrangian multiplier Γ is updated through

(14) $$\Gamma^{\left(k+1\right)}=\Gamma^{\left(k\right)}+\mu^{\left(k+1\right)}\left\{\begin{array}{c} P^{\left(k\right)}-L^{\left(k+1\right)}-E^{\left(k+1\right)} \end{array}\right\}$$

Similarly, the regularization parameter μ is updated through

(15) $$\begin{array}{ccc} \mu^{\left(k+1\right)} & = & min\left\{\begin{array}{c} \mu_{max},\rho \mu^{\left(k\right)} \end{array}\right\} \end{array}$$

where ρ is a carefully selected constant and $\mu_{max}$ is an adjustable parameter that influences the convergence of the proposed method. The remaining parameters are updated independently while keeping all other variables fixed.

As we invoked with affine transformation, we also achieved a new updating parameter Δτ. To make the new method easy to understand, the pseudocode is given in Algorithm 1.
**Algorithm 1** ADMM for RPCA with $L_{w,*}$ and $L_{2,1}$ norms.**Output** Data Matrix $N\in {ℜ}^{m\times n}$, $L^{0}\in {ℜ}^{m\times n}$, $E^{0}\in {ℜ}^{m\times n}$, $\Delta \tau^{0}\in {ℜ}^{p\times n}$, α, ρ
**While** not converged **Do** Update: $L^{\left(k+1\right)}$ using (7) Update: $E^{\left(k+1\right)}$ using (10) Update: $\Delta \tau^{\left(k+1\right)}$ using (13) Update: $\Gamma^{\left(k+1\right)}$ using (14) Update: $\mu^{\left(k+1\right)}$ using (15)**End while****Outputs**: L,E, Δτ

**Figure 1:**
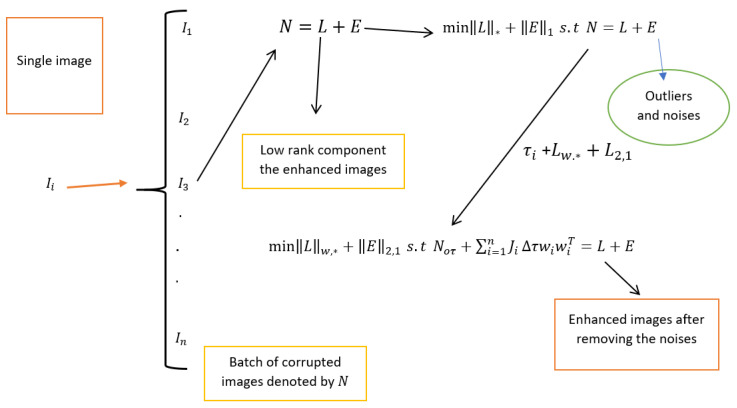
Flowchart of the robust PCA for retinal image decomposition.

In this paper, we evaluate the performance of the new method first using statistical measures through peak signal-to-noise ratio (PSNR) and structural similarity index measure (SSIM). We further verify the generalizability of the proposed method using the Pearson correlation coefficient (PCC) and Visual Information Fidelity (VIF) index based on on EyeQ, Kaggle, and High-Resolution Fundus (HRF) retinal image datasets, each containing its own degraded and ground truth images. In each experimental simulation, we first consider the degraded retinal images, then apply the new method to these images to achieve enhanced images. Next, we compare the enhanced images obtained through the proposed method with the ground truth. Finally, we compare the robustness of our method with existing methods. All experimental simulations are performed in MATLAB. Additionally, we consider regularization parameters, including $\rho =3\times 10^{-3}$, $\mu =3\times 10^{-10}$ and λ=4. For an easy understanding of the procedure of the proposed method, we support with the diagrammatic image representation shown in Figure 1.

### 2.4. Numerical Evaluation Criterion

The two popular criteria mainly used as quantitative evaluation indicators are the peak signal-to-noise ratio (PSNR) [85] and the structural similarity index measure (SSIM) [86,87].

The quality of retinal image enhancement by the proposed method is also validated using SSIM [86,87], which is given by

(16) $$\mathrm{SSIM}\left(f,\hat{f}\right)=\frac{\left(2\mu_{f}\mu_{\hat{f}}+C_{1}\right)\left(\left(2\sigma_{f}\sigma_{\hat{f}}+C_{2}\right)\right)}{\left(\mu_{f}^{2}+\mu_{\hat{f}}^{2}+C_{1}\right)\left(\sigma_{f}^{2}+\sigma_{\hat{f}}^{2}+C_{2}\right)}$$

where $\mu_{\hat{f}}$, $\sigma_{f}^{2}$, $\sigma_{\hat{f}}^{2}$ are the mean and variance of the ground truth and enhanced retinal images.

The Pearson correlation coefficient PCC [88] between the ground truth image *f* and the enhanced image $\hat{f}$ is given by:

(17) $$\mathrm{PCC}=\frac{\sum_{i=1}^{n}\left(f_{i}-\bar{f}\right)\left({\hat{f}}_{i}-\bar{\hat{f}}\right)}{\sqrt{\sum_{i=1}^{n}{\left(f_{i}-\bar{f}\right)}^{2}}\sqrt{\sum_{i=1}^{n}{\left({\hat{f}}_{i}-\bar{\hat{f}}\right)}^{2}}}$$

where:

(18) $$\bar{f}=\frac{1}{n}\sum_{i=1}^{n}f_{i}$$

(19) $$\bar{\hat{f}}=\frac{1}{n}\sum_{i=1}^{n}{\hat{f}}_{i}$$

The Visual Information Fidelity (VIF) [89] index between the ground truth image *f* and the enhanced image $\hat{f}$ is given by:

(20) $$\mathrm{VIF}=\frac{\sum_{i=1}^{N}\log_{2}\left(1+\frac{\sigma_{f_{i}}^{2}}{\sigma_{v_{i}}^{2}}\right)}{\sum_{i=1}^{N}\log_{2}\left(1+\frac{\sigma_{f_{i}-{\hat{f}}_{i}}^{2}}{\sigma_{v_{i}}^{2}}\right)}$$

where $f_{i}$ is the *i*-th block of the ground truth image *f*, ${\hat{f}}_{i}$ is the *i*-th block of the enhanced image $\hat{f}$, $\sigma_{f_{i}}^{2}$ is the variance of the i-th block of the ground truth image *f*, $\sigma_{f_{i}-{\hat{f}}_{i}}^{2}$ is the variance of the difference between the *i*-th block of the ground truth and enhanced images, and $\sigma_{v_{i}}^{2}$ is the noise variance in the *i*-th block of the ground truth image.

## 3. Datasets

In this section, three various retinal image datasets are used to evaluate the performance of the new method described in Section 2.2. The first dataset is the Eye Quality (EyeQ) retinal images which is taken from https://github.com/HzFu/EyeQ (accessed on 16 June 2024). To see the effectiveness of the new method, we used two independent datasets (both the training and testing images). The second dataset consists of retinal images infected with cataracts and glaucoma taken from the https://www.kaggle.com/datasets/jr2ngb/cataractdataset Kaggle dataset (accessed on 16 June 2024). The third dataset is taken from the High-Resolution Fundus (HRF) Image Database https://www5.cs.fau.de/research/data/fundus-images/ (accessed on 16 June 2024). We conducted comprehensive simulations across scenarios where ground truth, considered clean images, were available (full-reference assessment) for three distinct datasets to validate the generalizability of our method.

To see the efficacy of the new approach, we compared it with several existing approaches. Specifically, we considered HEM by [41], HIEA by [44], LLIE by [42], and one low-rank sparse method, TLLR by [64]. The methods we compared our proposed approach with are HEM by [41], TLLR by [64], LLIE by [42], and HIEA by [44]. In the upcoming subsections, we will delve into the datasets utilized, the numerical evaluation criteria employed, and the findings from our medical image analyses.

### 3.1. EyeQ Retinal Image Data

The Eye Quality (EyeQ) Assessment Dataset is a re-annotated subset of the EyePACS dataset, created for fundus image quality evaluation. The EyeQ dataset [7,90,91] consists of 28,480 training and 15,128 testing retinal images. The original retinal image dataset is manually labeled into three quality levels: good, usable, and reject. First, we considered 10 in low-quality images from the training dataset and 10 from the testing images, which are independent of the training dataset, to see the effectiveness of a novel approach in enhancing the low-quality images. This dataset encompasses the ground truth and degraded images, where the ground truth refers to the normal high-quality retinal images, while the degraded images, also called the low-quality retinal images, are simulated from the ground truth using light disturbance, image blurring, and retinal artifacts as outlined in [7,90,91]. The first two experiments predominantly focus on retinal image enhancement considering 10 true color degraded retinal images with a size of 800×800 pixels from the training dataset, while the second has a size of 256×256 pixels from the testing set; we considered this to assess the performance of the proposed method through visualization and numerical analysis.

### 3.2. Kaggle Cataract Retinal Image Data

The Kaggle dataset is another commonly used resource for evaluating the performance of the proposed method described in Section 2.1. The EyePACS dataset on Kaggle contains high-resolution retinal images used for cataract and other eye disease research. It includes thousands of annotated images, supporting diagnostic algorithm development. This dataset aids advancements in automated disease detection. Researchers use EyePACS to improve cataract diagnosis and eye care. This dataset contains retinal images that show symptoms of cataracts, glaucoma, and other diseases affecting human eyes. The cataract retinal images used for this analysis are taken from https://www.kaggle.com/datasets/jr2ngb/cataractdataset dataset (accessed on 16 June 2024), which consists of approximately 3500. The main focus of the final simulation results relies on the cataract retinal images, each having a size of 2464 × 1632 pixels.

### 3.3. High-Resolution Fundus Retinal Image Data

To verify the performance of the proposed method, we also considered the High-Resolution Fundus (HRF) dataset, a meticulously curated collection of retinal images tailored for developing and evaluating algorithms in medical image analysis. This dataset has three subjects: the first is the normal retinal images, the second is the retinal images infected with glaucoma, and the last one is retinal images with diabetic retinopathy, each with dimensions of 3304 × 2336 pixels. Its objective is to advance ophthalmology by considering high-quality images. It is also essential in the implementation of the new development of methods for detecting retinal diseases like diabetic retinopathy, macular degeneration, and glaucoma. The dataset includes 45 images, with 15 healthy retinas, 15 with diabetic retinopathy, and 15 with glaucoma. The high resolution of these images enables the precise identification of critical features such as blood vessels, optic discs, and lesions, which are essential for detecting conditions like diabetic retinopathy, glaucoma, and age-related macular degeneration. By utilizing the HRF dataset, we were able to implement the performance of the novel method compared with the state-of-the-art methods, enhancing our understanding of retinal diseases and advancing the development of automated diagnostic tools. The summary of all the datasets considered for retinal image data analysis is provided in Table 1.

## 4. Results

In this section, we aim to present experimental simulations of the new method compared with state-of-the-art methods such as HEM [41], TLLR [64], LLIE [42], and HIEA [44]. Initially, we conducted retinal image analysis using both the testing and training datasets. Subsequently, we attempted to simulate enhancement based on cataract retinal images. Finally, we conducted experimental simulations based on HRF diabetic retinopathy images.

### 4.1. Degraded Retinal Image Data Analysis

**Figure 2:**
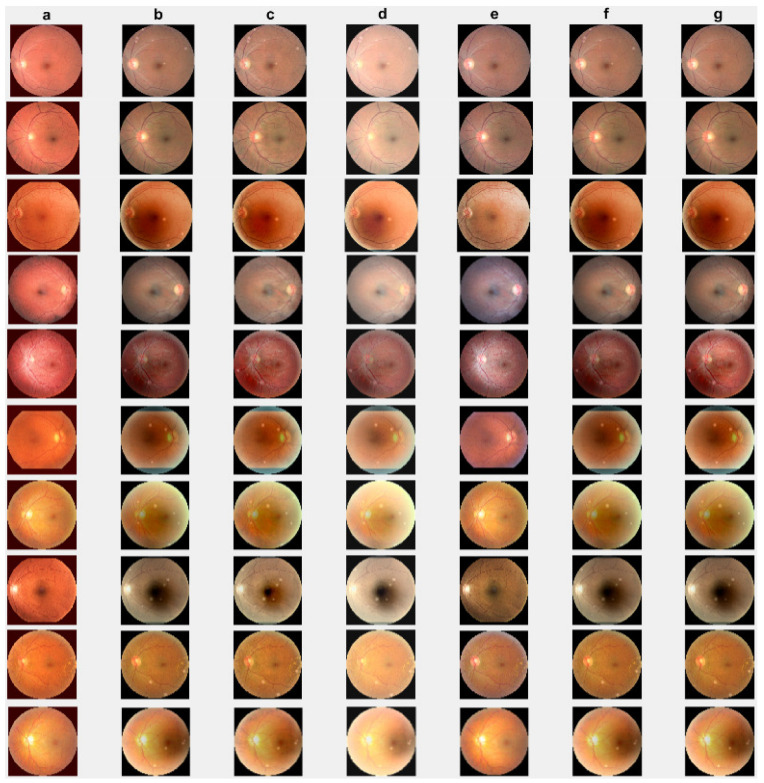
Degraded retinal image enhancement (training dataset): (**a**) HEM [41]; (**b**) TLLR [64]; (**c**) LLIE [42]; (**d**) HIEA [44]; (**e**) ours; (**f**) degraded image, and (**g**) ground truth.

**Figure 3:**
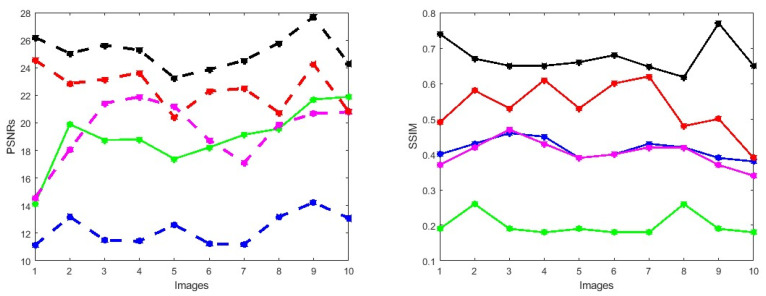
PSNRs and SSIMs obtained from the ground truth and enhanced images (training retinal degraded retinal images) computed by HEM [41] (blue color); TLLR [64] (green color); LLIE [42] (magenta color); HIEA [44] (red color) and ours (black color).

First, we conduct simulations on degraded retinal images taken from the training dataset as in [7,90,91]. In this experiment, 10 degraded retinal images with size 800×800 pixels are considered. As a visualization, some of the improved retinal images based on the above methods are given in Figure 2, in which our novel method, shown in Figure 2e, better enhances the degraded retinal images as compared to the state-of-the-art methods [41,42,44,64]. The values of the PSNR and SSIM based on the individual images are illustrated in Figure 3, from which we note that the new approach is relatively better to improve the low-quality individual original retinal images. To validate the performance of the proposed method, we employ PSNR and SSIM. HIEA [44] has better performance than HEM, shown by [41], as HIEA [44] requires a median filter, which was employed for image denoising, which boosts the effectiveness of the new approach as compared with LLIE developed by [42]. This result resembles the results given in Table 2, and further confirms that the new method is more resilient to the degradation factors, which means that the new approach outperformed the four competitors in all evaluation metrics.

Furthermore, we incorporate 10 degraded images from the EyeQ test dataset, which has been commonly referenced in prior studies [7,90,91], to simulate additional light interference, image blurring, and artifacts.

**Figure 4:**
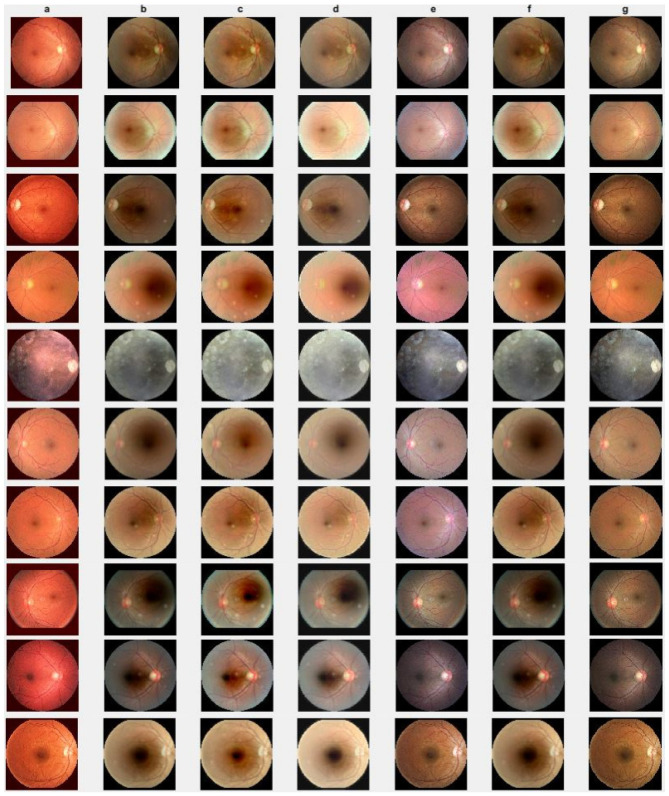
Degraded retinal image enhancement (testing dataset): (**a**) HEM by [41]; (**b**) TLLR by [64]; (**c**) LLIE by [42]; (**d**) HIEA by [44]; (**e**) ours; (**f**) degraded image, and (**g**) ground truth.

**Figure 5:**
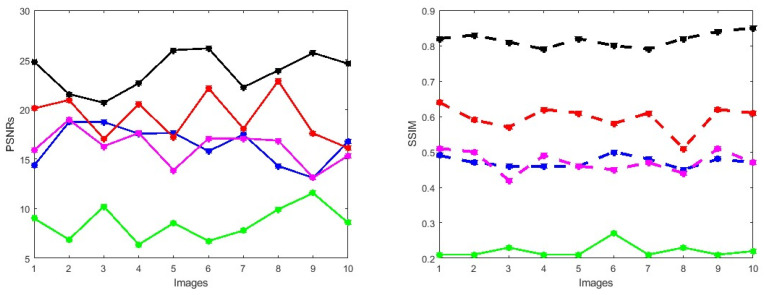
PSNRs and SSIMs obtained from the ground truth and enhanced images (testing retinal degraded retinal images) computed by HEM [41] (blue color); TLLR [64] (green color); LLIE [42] (magenta color); HIEA [44] (red color) and ours (black color).

We check the performance of the proposed method to enhance the degraded images through visualization and numerical measures using the PSNR and SSIM between the degraded low-quality images and their high-quality counterparts. As illustrated, Figure 4e shows some visual images enhanced by the proposed method compared with existing methods. These images demonstrate that the proposed method significantly enhances degraded retinal images, bringing them closer to the ground truth. The enhanced images exhibit clearer visual quality by effectively removing light disturbance, image blurring, and retinal artifacts. To further verify the performance of the new approach based on individual retinal images, we compare it using the PSNR and SSIM with existing methods as shown in Figure 5. LLIE [42] outperforms HEA [41] by considering multi-resolution branches for a better understanding of different levels of local and global context, thus mitigating the influence of outliers and noise. HIEA [44] surpasses all three existing methods, as it is a hybrid algorithm incorporating a median filter for image denoising. Figure 4e demonstrates that the new method achieves the best performance [41,42,44,64]. The summary values of the PSNR and SSIM for ten retinal images achieved by the proposed method along with existing methods are shown in Table 3. From this table, we can see that HIEA by [44] produces the second-best performance. This aligns with the results presented in Figure 4 and further justifies that the proposed method better enhances the degraded retinal images as compared with existing methods. The summary values of the PSNR and SSIM for ten retinal images achieved by the proposed method along with existing methods is given in Table 3, from which we can observe that HIEA by [44] produces the second-best performance. The performance of the proposed method is evaluated, taking more retinal images, and it is confirmed that the performance is better than the existing methods. This is because HIEA combines median filtering to reduce variation and combines it with deep learning to minimize the $L_{2,1}$ norm of the sparse error. We can also observe from Table 3 that the new approach still outperforms all existing methods. It achieves this by including affine transformations and utilizing the $L_{2,1}$ and $L_{w,*}$ norms to render more robust degraded retinal image recovery. The visual enhancement of degraded retinal images shown in Figure 4e and Figure 5 is more consistent with the numerical evaluation analysis provided in Table 3. This is because the new method incorporates a set of affine transformations and employs the $L_{2,1}$ and $L_{w,*}$ norms to simultaneously align and enhance the retinal images. This enables the new method to reduce the influences of outliers, heavy sparse noise, occlusions, light transmission issues, image blurring, retinal artifacts, and illuminations.

**Figure 6:**
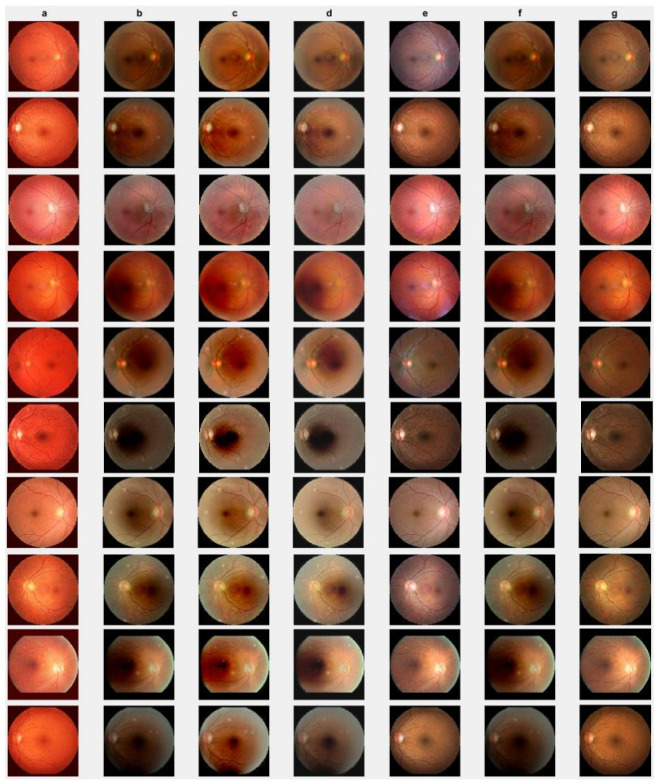

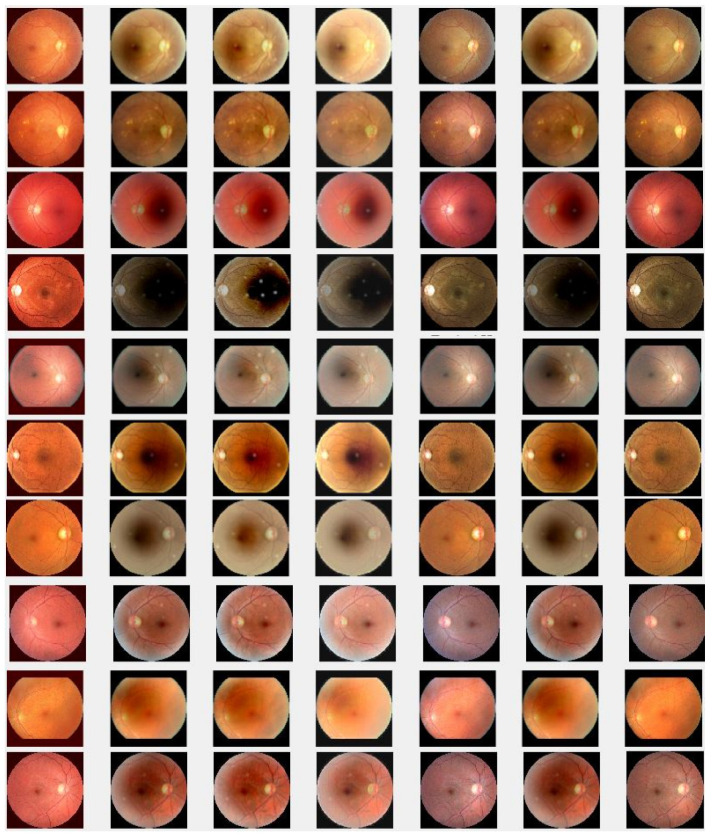

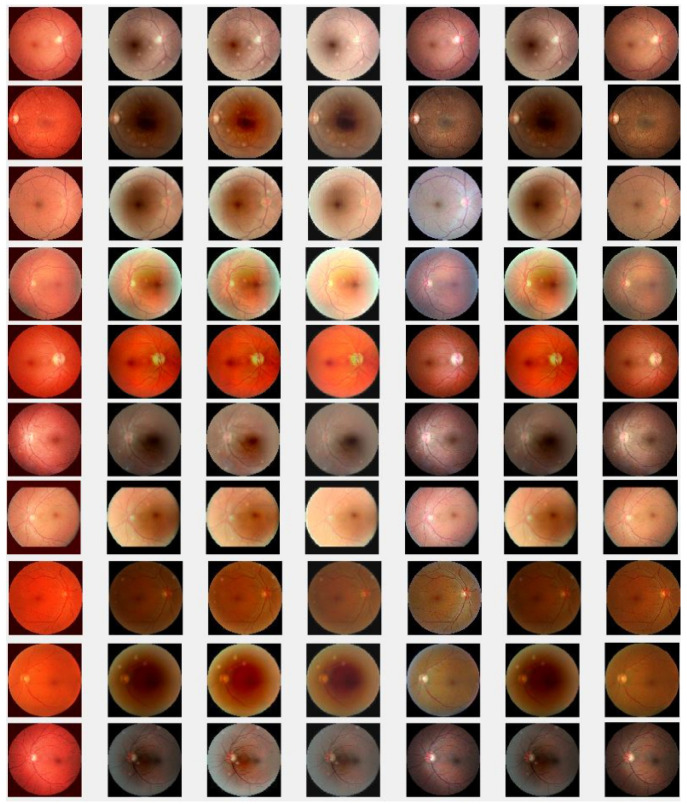

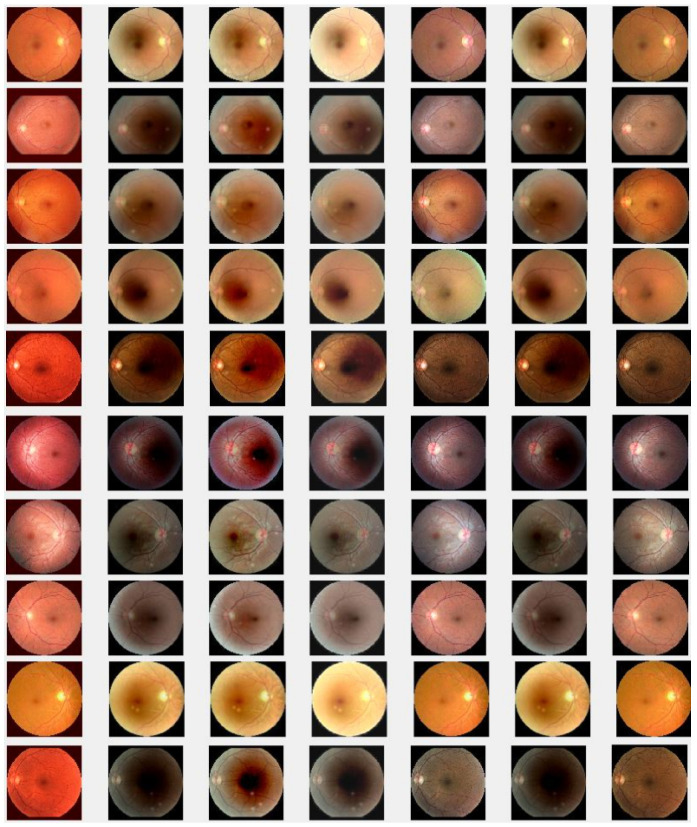
Degraded retinal image enhancement (training dataset): (**a**) HEM [41]; (**b**) TLLR [64]; (**c**) LLIE [42]; (**d**) HIEA [44]; (**e**) ours; (**f**) degraded image and (**g**) ground truth.

**Figure 7:**
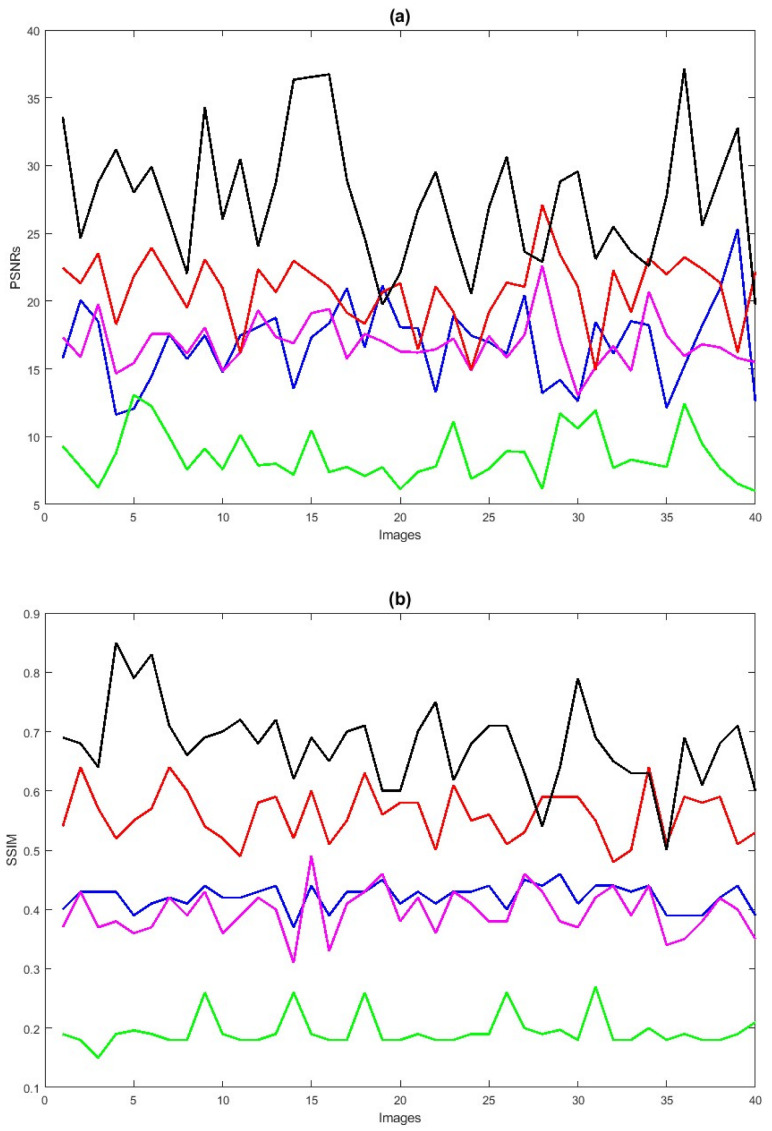
PSNRs (**a**) and SSIMs (**b**) vs. degraded 40 retinal images. HEM [41] (blue color); TLLR [64] (green color); LLIE [42] (magenta color); HIEA [44] (red color); ours (black color).

Next, we also conduct simulations on a more challenging set of 40 large samples of low-quality degraded retinal images with size 256×256, sourced from [7,90,91], to verify the performance of the proposed method in enhancing these retinal images as shown in Figure 6e, demonstrating superior performance compared to existing methods. We also verify the effectiveness of the proposed method by taking 40 retinal images and confirm that the new method is better compared to the baselines, both through visualization and numerical analysis. Subsequently, the comparison of PSNRs and SSIMs is summarized in Table 4, revealing that LLIE [42] outperforms HEM [41] and TLLR [64]. Meanwhile, our proposed method outperforms the existing methods due to its incorporation of affine transformations, $L_{w,*}$ and $L_{2,1}$ norms. Statistically, the proposed method demonstrates superiority over the other four competitors [41,42,44,64] in terms of PSNR and SSIM as shown in Table 4. The PSNRs and SSIMs for individual images are given in Figure 7, indicating that the performance of the proposed method surpasses that of the existing methods. This improvement is attributed to the incorporation of affine transformation and $L_{w,*}$ and $L_{2,1}$ norms to further denoise the degraded retinal images.

**Table 3:** Comparison of methods by the PSNR and SSIM (ground truth and enhanced images).

Methods	Based on Figure 4		Based on Figure 8	
	PSNR	SSIM	PSNR	SSIM
HEM [41]	15.78	0.43	12.28	0.52
TLLR [64]	11.40	0.24	18.94	0.50
LLIE [42]	16.73	0.39	19.44	0.55
HIEA [44]	21.57	0.57	22.52	0.65
Ours	27.87	0.72	25.15	0.78

### 4.2. Cataract Retinal Image Data Analysis

**Figure 8:**
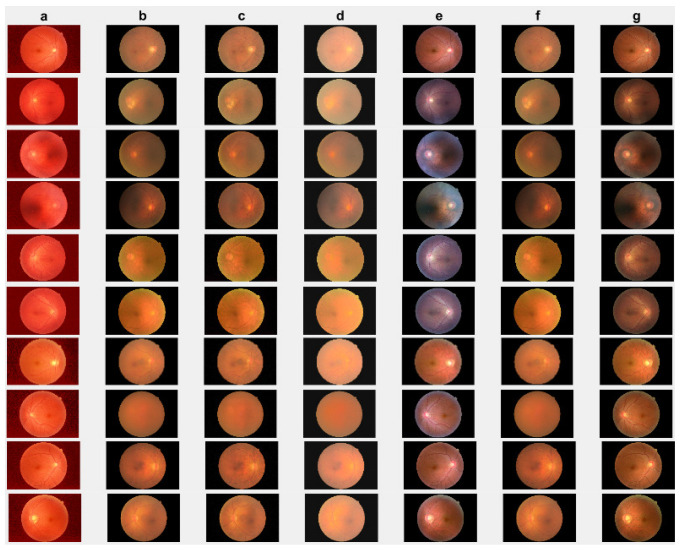
Cataract infected low-quality retinal image enhancement: (**a**) HEM [41]; (**b**) TLLR [64]; (**c**) LLIE [42]; (**d**) HIEA [44]; (**e**) ours; (**f**) degraded image, and (**g**) ground truth.

**Figure 9:**
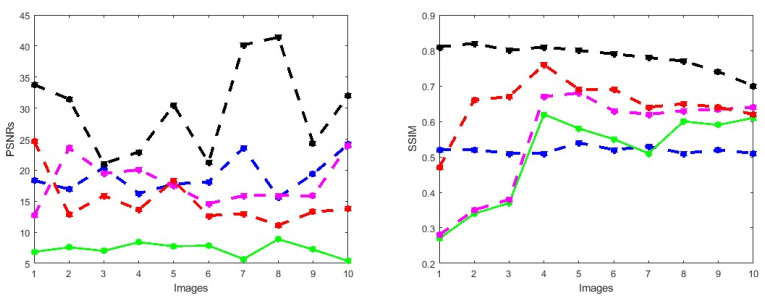
PSNRs and SSIMs obtained from the ground truth and enhanced images (cataract retinal images) computed by HEM [41] (blue color); TLLR [64] (green color); LLIE [42] (magenta color); HIEA [44] (red color) and ours (black color).

In this section, we present the results of the proposed method compared with existing methods [41,42,44,64] based on more challenging and high-dimensional cataract retinal images from the Kaggle dataset. In this simulation, 10 retinal images with cataract with size of 2464 × 1632 are considered. The visualization results show that the proposed method performs better in enhancing ten retinal images infected with cataracts, closely aligning with the ground truth as depicted in Figure 8e. To evaluate the performance of the proposed method compared with existing methods for individual retinal images, we compute PSNRs and SSIMs, from which we observe that the proposed method outperforms in enhancing cataract retinal images as depicted in the third row of Figure 9. The result of Table 3 is consistent with the results shown in image visualization. This improvement is attributed to the proposed method considering $\tau_{i}$, and the $L_{w,*}$ and $L_{2,1}$ norms. This is consistent with the results in Table 3, and further confirms that the proposed method is more resilient to outliers and heavy sparse noise.

### 4.3. HRF Image Database

**Figure 10:**
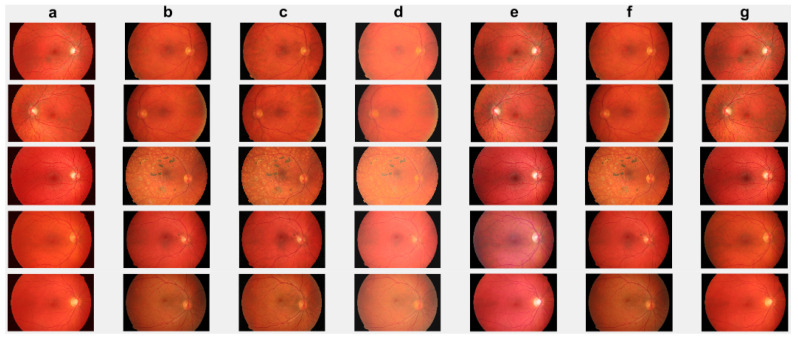
Diabetic retinopathy retinal image: (**a**) HEM by [41]; (**b**) TLLR by [64]; (**c**) LLIE by [42]; (**d**) HIEA by [44]; (**e**) ours; (**f**) degraded image and (**g**) ground truth.

**Figure 11:**
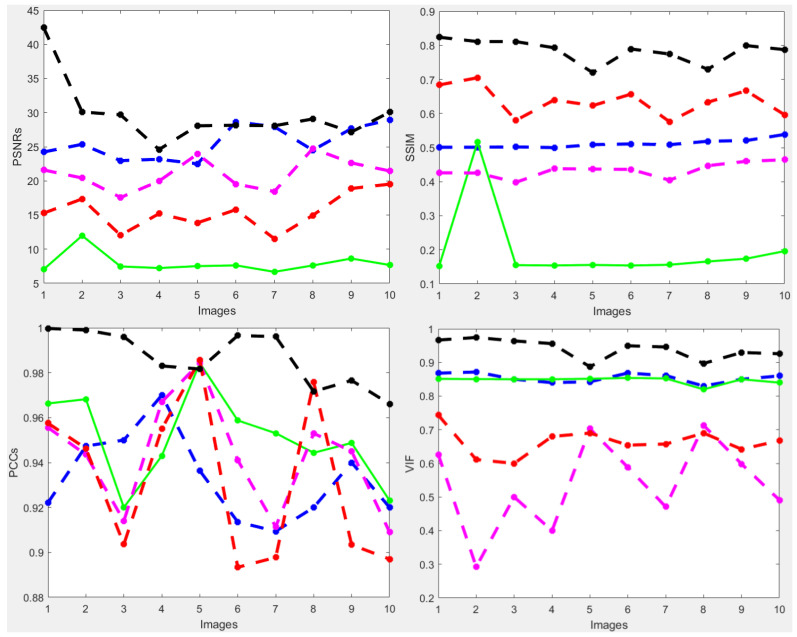
PSNRs and SSIMs obtained from the ground truth and enhanced images (HRF diabetic retinopathy) (first row). The values of PCCs and VIFs obtained from the ground truth and enhanced images (HRF diabetic retinopathy) (second row) computed by HEM [41] (blue color); TLLR [64] (green color); LLIE [42] (magenta color); HIEA [44] (red color) and ours (black color).

To further verify the performance of the new method, we also considered a complicated and high-dimensional dataset infected with various diabetic retinal images, with each having a size of 3304 × 1632 pixels, for which the visualization results are displayed in Figure 10. The result achieved by the novel method shown in Figure 10e is better in enhancing the quality of the diabetic retinal images compared with the state-of-the-art methods. This result is more consistent with the numerical simulations given in Table 5. The individual results using PSNRs, SSIMs, PCCs and VIFs are illustrated in Figure 11, in which the performance achieved by the proposed method is better compared to the existing methods.

## 5. Discussion and Conclusions

This work is dedicated to the enhancement of retinal images by the RPCA method [53,54] through $L_{w,*}$, $L_{2,1}$ and affine transformation, aimed at improving their robustness. The development of these approaches represents a significant contribution to the field of statistics in imaging, particularly in the realm of high-dimensional medical images. Existing methods, as referenced in [53,54], often lack robustness in the presence of gross errors and outliers within high-dimensional retinal images. In response to this challenge, this article presents a novel method developed to address these issues head on.

In this paper, we propose a novel method for enhancing low-quality retinal images, which is crucial for detecting cataracts and diabetic diseases in human eyes. To ensure robustness against anomalies such as light disturbances, image blurring, and retinal artifacts, the proposed new method combines $\tau_{i}$, and $L_{w,*}$ and $L_{2,1}$ norms into RPCA, thereby advancing the existing optimization techniques. The proposed method for enhancing low-quality retinal images is a multifaceted approach that integrates several mathematical frameworks to address the common challenges encountered in retinal imaging. The incorporation of $\tau_{i}$, $L_{w,*}$ and $L_{2,1}$ norms into Robust Principal Component Analysis (RPCA) represents a sophisticated novel strategy to enhance the robustness of the image processing algorithm.

The utilization of $\tau_{i}$ for image alignment through Taylor series expansion and Jacobian transformation provides a novel application of geometric principles in the context of retinal image processing. By iteratively updating the warp parameters, this technique aims to mitigate misalignments induced by factors such as eye movement or imaging artifacts. Moreover, the introduction of the $L_{2,1}$ norm serves to address sparse adverse effects within the retinal images, leveraging sparse regularization to effectively handle noise and outliers. This norm contributes to the alignment of retinal images by minimizing the impact of correlated samples and promoting consistency across the dataset. The incorporation of the weighted nuclear norm, denoted by $L_{w,*}$, introduces a nuanced approach to singular value regularization, where weights are assigned to individual singular values based on their significance in the image processing context. By assigning appropriate weights, this technique aims to preserve important features while suppressing noise and irrelevant variations, thereby enhancing the fidelity of the reconstructed images.

The validation of this proposed new method on two widely used public datasets demonstrates its efficacy in real-world scenarios and provides empirical evidence of its superiority over existing methods. The comparative evaluation highlights the potential of this new method to significantly enhance the quality of retinal images, which is crucial for accurate disease detection and diagnosis.

As future research, this work can be extended to incorporate truncated weighted nuclear norm regularization for image denoising and its integration into Tensor RPCA. By extending the proposed new method to handle more complex data structures and incorporating additional regularization parameters, this work has the potential to enhance the quality of the retinal image processing over the baselines.

## Figures and Tables

**Table 1:** Characteristics of retinal image datasets.

Dataset	Type	Size
EyeQ [7,90,91]	Retinal-Training	800×800 pixels
	Retinal-Testing	256×256 pixels
Kaggle	Cataract	2464×1632 pixels
HRF	Diabetic Retinopathy	3304×1632 pixels

**Table 2:** Comparison of methods by PSNR and SSIM (training dataset).

Methods	PSNRs	SSIMs
HEM [41]	19.06	0.47
TLLR [64]	7.26	0.22
LLIE [42]	18.10	0.47
HIEA [44]	22.50	0.60
Ours	24.33	0.76

**Table 4:** Comparison of methods by the PSNR and SSIM (testing dataset).

Methods	PSNR	SSIM
HEM by [41]	16.95	0.42
TLLR by [64]	10.45	0.37
LLIE by [42]	16.99	0.39
HIEA by [44]	20.82	0.56
Ours	27.17	0.68

**Table 5:** Comparison of methods by the PSNR, SSIM, PCC and VIF based on HRF dataset.

Methods	PSNR	SSIM	PCC	VIF
HEM [41]	24.78	0.50	0.93	0.86
TLLR [64]	6.97	0.21	0.95	0.85
LLIE [42]	20.51	0.43	0.95	0.47
HIEA [44]	14.92	0.65	0.94	0.69
Ours	28.006	0.79	0.987	0.949

## Data Availability

Applicable.

## Abbreviations

The following abbreviations are used in this manuscript:

MDPI	Multidisciplinary Digital Publishing Institute
ADMM	Alternating Direction Method of Multipliers
CLAHE	Contrast Limited Adaptive Histogram Equalization
LLIEM	Low Light Image Enhancement Method
RPCA	Robust Principal Component Analysis
PSNRs	Peak Signal-to-Noise Ratio
SSIMs	Structural Similarity Index
EyeQ	Eye Quality
HIEA	Hybrid Image Enhancement Algorithm
WNNM	Weighted Nuclear Norm Minimization
TLLR	Tensor Low-Rank Representation
VIF	Visual Information Fidelity
PCC	Pearson correlation coefficient
